# Considerations for biologics as front-line treatment in allergic diseases

**DOI:** 10.3389/fimmu.2025.1746790

**Published:** 2026-01-05

**Authors:** Akash Kothari, Lisa Hung, Julia E. M. Upton

**Affiliations:** 1 Temerty Faculty of Medicine, University of Toronto, Toronto, ON, Canada; 2 Division of Allergy and Immunology, Department of Medicine, Feinberg School of Medicine, Northwestern University, Chicago, IL, United States; 3 Division of Immunology and Allergy, Department of Paediatrics, Hospital for Sick Children, SickKids Food Allergy and Anaphylaxis Program, University of Toronto, Toronto, ON, Canada

**Keywords:** biologics, biomarkers, food allergy, immunotherapy, prevention

## Abstract

The therapeutic landscape of allergic diseases has been transformed by the advent of biologics targeting key immunologic pathways. These therapies offer disease-modifying potential across a spectrum of conditions including asthma, atopic dermatitis, eosinophilic esophagitis, chronic rhinosinusitis with nasal polyps, and food allergy. However, their high cost, limited long-term safety data in some populations, and evolving eligibility criteria raise critical questions about when biologics such as monoclonal antibodies are truly warranted, especially in the context of food allergy. Agents such as omalizumab have demonstrated efficacy in raising the threshold of allergic response with monotherapy and during oral immunotherapy, improving safety profiles and accelerating desensitization. Recent developments in pharmaceutical-grade food immunotherapy and adjunctive/alternative biologic use further complicate decision-making. This expanding therapeutic toolbox necessitates a critical reassessment of when biologics are justified as front-line in allergic diseases such as food allergy. Monoclonal antibodies, in particular, are usually reserved for step-wise treatment of other diseases. It is important to have ongoing assessments as to which new and upcoming treatment modalities should be first-line. As food allergy management becomes increasingly interventional, providers must weigh the benefits of biologic therapies against real-world feasibility, health economics, and patient preference among other alternatives. Here, we discuss the current management of IgE-mediated food allergy as well as emerging therapeutics including immunotherapies and biologics. We evaluate the positioning of omalizumab in food allergy, compared with other biologics and off the shelf food-based approaches used in food allergy, and discuss clinical and research implications.

## 1 Introduction

In recent years, there has been a shift in the treatment of allergic diseases, from avoiding triggers and mitigating symptoms to actively increasing the threshold of reaction for food allergy. This is in large part due to the introduction of biologics and alternative treatment options for atopic disorders. Monoclonal antibodies are now used to treat a variety of allergic diseases including asthma, atopic dermatitis (AD), eosinophilic esophagitis (EoE), chronic rhinosinusitis with nasal polyps (CRSwNP), and food allergy (1, 2). As more treatment options become available, the question of how treatments should be positioned increases in relevance. For food allergy, it is now widely accepted that reaction threshold can be modified through allergen immunotherapy, including off the shelf foods and standardized pharmaceutical flour (peanut), or the use of the biologic, omalizumab. However, both options are limited by availability, cost, safety profile and efficacy.

As peanut allergies are one of the most common food allergies in the world, the Food and Drug Administration (FDA) approval of AR101, a pharmaceutical-grade peanut flour for oral immunotherapy in 2020, was a milestone moment for the field (3). It was the first FDA approved treatment for peanut allergies and marked a turning point for the way peanut allergies are perceived and treated. The recent approval of omalizumab as a monotherapy in 2024 for multi-food allergy was also a pivotal moment (1). This therapeutic increases the threshold of allergic reaction irrespective of the specific allergen, providing a potential treatment option for individuals with multiple food allergies. Both of these treatments are positioned as first-line therapies, but differ important ways. Other biologics like dupilumab have shown significant effects for other atopic disorders like EoE or AD as a monotherapy (2, 4). For food allergy, dupilumab has not been proven to be a strong candidate and does not have FDA approval for use in this context. However, it may be useful in allergen immunotherapy as an adjunct therapy given its other indications (5, 6).

Several factors are involved in choosing to pursue one treatment over another. Food allergies, like many disorders, are highly heterogeneous. Reaction threshold and severity range widely between individuals, as well as food-allergy related anxiety and effects on quality of life. Each treatment modality for food allergy is a long-term commitment, therefore the decision to proceed with a particular treatment must be made with each individual patient in mind.

This article provides an overview of the current landscape of food allergy treatment, highlighting the benefits and limitations of different strategies and provides commentary for when certain options such as biologics may be most suitable.

## 2 Allergen immunotherapy current landscape

### 2.1 Oral immunotherapy

Oral immunotherapy (OIT) is the most common form of allergen immunotherapy for foods (7). It involves the gradual ingestion of food allergens below the reaction threshold, increasing the quantity over an extended period until a maintenance dose is reached. The goal of OIT is to increase the patient’s reaction threshold and desensitize the patient to their culprit food, to both reduce reactivity and severity of allergic responses. For most patients, desensitization can be achieved, but the maintenance dose is required for many years or possibly indefinitely to retain this protection. OIT is generally not a curative treatment, but in some individuals, it can induce a period of non-reactivity termed sustained unresponsiveness or remission, where the patient can maintain protection from the allergen despite halting the maintenance dose. Achieving remission seems most likely in young children (8).

Before AR101 was approved by the FDA, allergists were providing OIT to patients through the use of extracts or commercially available foods and continue to do so given its proven efficacy (9). Patients on OIT report decreased reaction severity and increased quality of life during and after protocol completion (10, 11). OIT for multiple allergies can also be done using mixed extracts, providing an option for multi-food allergic individuals. However, OIT targeting a specific food does not typically appear to affect other allergens if the individual is multi-food allergic unless the allergens have a high degree of cross-reactivity (12).

Patients often report mild to moderate adverse reactions while on OIT, particularly gastrointestinal symptoms including stomach pain and vomiting, as well as an increased risk of anaphylaxis during the first few years. Additionally, OIT is particularly sensitive to changes in physiology and barrier function, resulting in additional restrictions to patient behavior before and after allergen ingestion. These restrictions (e.g. hot showers, exercise, alcohol consumption), also known as cofactors, have been shown to decrease reaction threshold and increase the risk of a reaction even during maintenance doses (13).

Despite numerous studies looking into optimal timing and dosing, no standardized protocol has been reached that works for all allergens or individuals. The ideal protocol may be influenced by a variety of patient intrinsic factors including food specific IgE levels, reaction threshold, comorbidities, age, patient preference and many others. Recent studies have shown that maintenance doses may not need to be very high to induce protection from accidental exposures while also reducing the risk of adverse effects (14, 15).

### 2.2 OIT with pharmaceutical-grade biological products

AR101 has proven the importance and utility of pharmaceutical-grade food products for OIT. The dosing protocol of pre-weighed and aliquoted capsules of peanut flour provides a consistent method of allergen ingestion. This standardized dosing may be particularly significant for individuals with very severe peanut allergies, and during the initial stages of treatment where minute differences in dosage may be potentially life-threatening. The capsules also prevent cross-contamination, and the highly refined production requirements provide an additional layer of standardization and traceability. However, due to the resources required to produce and distribute this product, the availability of pharmaceutical-grade food products like AR101 are often limited by cost and health care coverage (16, 17). OIT can also be administered using home-made or store-bought commercial products like peanut butter or an equivalent for other allergens (18). While this provides a much more cost effective and convenient option, issues of dose standardization are particularly relevant, especially for smaller amounts. Individuals well trained in measuring doses and/or those with a higher reaction threshold can find this a convenient and cost-effective option (19). Furthermore, many countries do not have access to standardized, pharmaceutical grade formulations and alternative formats are the only option.

### 2.3 SLIT and EPIT overview

Sublingual immunotherapy (SLIT) and epicutaneous immunotherapy (EPIT) involve absorption of much smaller quantities of allergen through the sublingual mucosa and skin, respectively. SLIT involves the absorption of allergen via drops or tablets under the tongue, whereas EPIT utilizes patches of allergen on the skin. These modalities appear to reliably provide protection at doses expected to allow for safe accidental exposures and beyond (20, 21). Mild to moderate local site reactions are commonly reported, with epinephrine rarely required (22). For EPIT, skin reactions tend to decrease with continued use over time (23, 24). For both modalities the side effect rate is low enough that avoiding cofactors/life-style restrictions are not advised (25). Treatment adherence for EPIT is relatively high whereas it varies more widely for SLIT between studies (23, 26). Like OIT, younger age at the start of both SLIT and EPIT improves outcomes for patient desensitization and overall treatment efficacy (23, 27). Variation between protocols have illustrated differences between routes of administration as well as dose effects in comparison to OIT, with lower doses of OIT potentially providing suitable protection with fewer side effects (15).

### 2.4 SLIT and EPIT pharmaceutical products

For peanut EPIT, the VIASKIN patch has been through extensive investigation. Multiple clinical trials utilizing the patch to treat peanut allergy have shown efficacy in desensitization via increased reaction eliciting dose with good safety profiles in children (28, 29). Recent findings using the VIASKIN patch with 250ug peanut (VP250) for peanut EPIT in children aged 1–3 years old at enrollment have shown increased reaction thresholds and desensitization after 2 years of treatment with limited adverse effects in the phase 3 EPITOPE Trial (21). Atopic comorbidities do not appear to affect the safety or efficacy of peanut EPIT (30).

Recent studies utilizing SLIT for the treatment of food allergy have been using commercially available food products, expanding the accessibility and cost effectiveness of this treatment (20, 25). Using SLIT prior to OIT has also been shown to safely introduce allergen immunotherapy to higher risk allergic children, with further studies under investigation (NCT05440643) (31).

## 3 Biologics in food allergy: mechanisms and indications

### 3.1 Biologics as a monotherapy

Omalizumab is a humanized, recombinant monoclonal antibody targeting immunoglobulin E. It increases the threshold of reactivity by reducing the amount of circulating allergen-specific IgE. As a monotherapy, omalizumab has shown great efficacy increasing the amount of allergen tolerated during double-blind placebo-controlled food challenges (32–35). It was initially approved by the FDA for the treatment of severe allergic asthma in 2003 and based on phase III trials including OUtMATCH showing both efficacy and safety, was approved to treat multi-food allergy in the United States in 2024, marking the first biologic available for use in food allergy (1). The treatment involves either biweekly or monthly injections either in clinic or at-home with no other interventions aside from continued avoidance of the allergen. Used in this way, omalizumab is an additional safety measure to increase the threshold of allergic response and reduce the risk of anaphylaxis for severe food allergy.

Dupilumab is a monoclonal antibody targeting the receptor for interleukin-4 and interleukin-13. It was first approved by the FDA for the treatment of severe AD in 2017 and has since been extended for the treatment of asthma, CRSwNP, EoE, prurigo nodularis, chronic obstructive pulmonary disease, chronic spontaneous urticaria, and bullous pemphigoid. Also administered as a subcutaneous injection, dupilumab has proven very effective for the treatment of the indicated disorders. As dupilumab targets the type 2 pathway and has been associated with a marked decrease in allergen specific IgE levels, some studies have suggested it may be effective for treating food allergies (36, 37). However, dupilumab as a monotherapy for food allergy had minimal effect on desensitization (6).

### 3.2 Biologics as an adjunct to allergen immunotherapy

The use of omalizumab to enable faster, safer up-dosing for OIT has been a key question of multiple clinical trials. Numerous trials have attempted to answer whether the risk of an allergic reaction can be reduced while also reintroducing the allergen back into the diet (12, 38). However, long-term data is still forthcoming and biologics beyond omalizumab have not demonstrated any clear effectiveness (e.g., etokimab, ligelizumab - NCT04984876, NCT05678959) (39, 40). Further investigative trials may yet provide further insight into biologics for allergen immunotherapy (NCT05432388, NCT05069831, NCT04045301).

Dupilumab as an adjunct to OIT showed mild improvements over OIT alone with effects lost post-treatment (5). It is unclear whether the use of dupilumab in addition to omalizumab may be more effective than omalizumab as a monotherapy, or if the use of dupilumab in patients with atopic comorbidities, such as AD with FA would be more beneficial (41).

### 3.3 Broader context: biologics in other diseases

The current indication for omalizumab to treat food allergy is extensive, with the only criteria being age ≥1 year and dosage according to total serum IgE (IU/mL) and weight. Importantly, there is no need to try or fail any prior management. However, given that there are alternative, disease modifying treatments available and that some allergic children outgrow their allergy with age, it is unclear if use of a biologic as a first-line treatment is the best option. This positioning of omalizumab differs from all its other indications where the FDA approval indicates another treatment needs to be tried first (**Table 1**).

**Table 1:** Omalizumab approved indications biologic medications approved for food allergy as of 2025.

Atopic condition	FDA indication / criteria	Position relative to other therapy
All FDA approved Omalizumab Indications (42)		
IgE-mediated Food Allergy	Reduction of allergic reactions (including anaphylaxis) from accidental exposure in patients ≥ 1 year with IgE-mediated food allergy.	First-line risk-reduction (no prior therapy failure required)
Moderate-to-Severe Allergic Asthma	Patients ≥ 6 years with positive test to perennial aeroallergen, and asthma not controlled with inhaled corticosteroids ± LABA.	Step therapy: after standard controller failure
Chronic Spontaneous Urticaria	Adults/adolescents ≥ 12 years with hives not controlled by H1 antihistamines.	After failure of high-dose antihistamines
Chronic Rhinosinusitis with Nasal Polyps (CRSwNP)	Adults ≥ 18 years with inadequate response to intranasal corticosteroids.	Add-on maintenance therapy
Food allergy related/adjacent indications for other biologics (excluding asthma)		
IgE-mediated Food Allergy (43)	Pharmaceutical-grade peanut flour is approved for age 1 to 17 years	No formal requirement to fail prior therapy is stated in the FDA labelling or approval documents
Eosinophilic esophagitis (44)	Dupilumab is approved for patients ≥ 1 year and ≥ 15kg	Step therapy: PPI + topical therapies first (or if PPI + topical therapies are not advisable)
Atopic Dermatitis (44)	Dupilumab is approved for patients ≥ 6 months of age	Step therapy: topical therapies first (or if topical therapies are not advisable)

LABA, Long-acting beta-agonists; PPI, proton pump inhibitor.

Alternative therapies now available for the treatment of food allergy include OIT, SLIT and EPIT which are immunomodulating, and there is increasing evidence that early food-based interventions may change the course of food allergy persistence (45, 46). The only pharmaceutical grade, FDA approved food-based therapy is also front-line (**Table 1**) including those from 1–17 years of age.

While no other food allergy indications exist at this time for biologics, given that food allergy is typically concomitant with other atopic indications, biologics are an appealing option to manage these comorbid diseases if patients meet eligibility criteria. This may provide an advantage to patients, where a single biologic may be useful as a monotherapy for multiple conditions or as an adjunctive therapy if other first-line treatments are available. In the setting of asthma and AD, only moderate-severe phenotypes qualify for biologics after inadequate control through other modalities (47, 48). Dupilumab has indications for AD after failure of topical treatments and is front-line for EoE (**Table 1**). Omalizumab and pharmaceutical peanut flour both have the same regulatory designation as biologics despite being a monoclonal antibody and a standardized food. The front-line indications for both of these medications which differ so extensively show the need for treatments for food allergy. These medications should further move the management from passive avoidance. Given that food immunotherapy has potentially sustained effects including remission, omalizumab as a front-line agent is worth further evaluation.

## 4 Pros and cons of omalizumab

### 4.1 Benefits

Omalizumab provides a relatively non-invasive, safe and effective treatment option. It requires minimal effort, either biweekly or monthly injections, which can be given at home, with no other required intervention for improved protection. Short-term adverse events are mild, generally injection-site reactions like redness and pain. Due to its mechanism of action, it is allergen-agnostic and can be used to treat multiple food allergies at once. Long-term usage of omalizumab (>9 years) for the treatment of allergic asthma has shown favorable safety profiles and continued efficacy (49, 50). Omalizumab protects against the episodic but potentially catastrophic risk of anaphylaxis by decreasing the risk of reaction.

While allergen immunotherapy (AIT) is growing in use, it is still marred by a high risk of adverse events and reactions due to direct exposure to the allergen. Cofactors encountered during daily life can also increase risk of reactions. This causes lower adherence to the therapy and may affect efficacy if doses are missed or decreased. The use of biologics as an adjunct to AIT lowers the risk of adverse reactions, enhancing safety profiles while also potentially allowing for faster desensitization and up-dosing.

### 4.2 Limitations

Access to omalizumab for food allergies is highly dependent on location and health insurance coverage. Biologics require prescriptions and ongoing access to a specialist, as well as the continuous costs of the medication itself, limiting availability.

Long-term administration is required to sustain benefits, and any changes in dosing either due to loss of availability or illness may affect their protective effects. In this regard, due to the relatively new approval of omalizumab for food allergies, the long-term experience for this indication is limited. Omalizumab is not currently intended to be a curative treatment even with ongoing use; patients on this biologic are advised to continue avoidance.

For omalizumab, there are no checkpoints aside from initial IgE measurements for eligibility qualification. IgE measurements following administration no longer provide diagnostic value, and basophil activation tests or skin prick tests are not requirements to assess for efficacy. It is known that there is heterogeneity in response, but these patients also have no method of understanding their level of protection at any point during treatment. While it is more than likely that omalizumab is providing a significant level of protection from accidental exposure for most patients, there may be some that are not receiving the protective effects while engaging in risk-taking behavior. It is assumed this protection would be lost upon discontinuing treatment.

**Figure 1:**
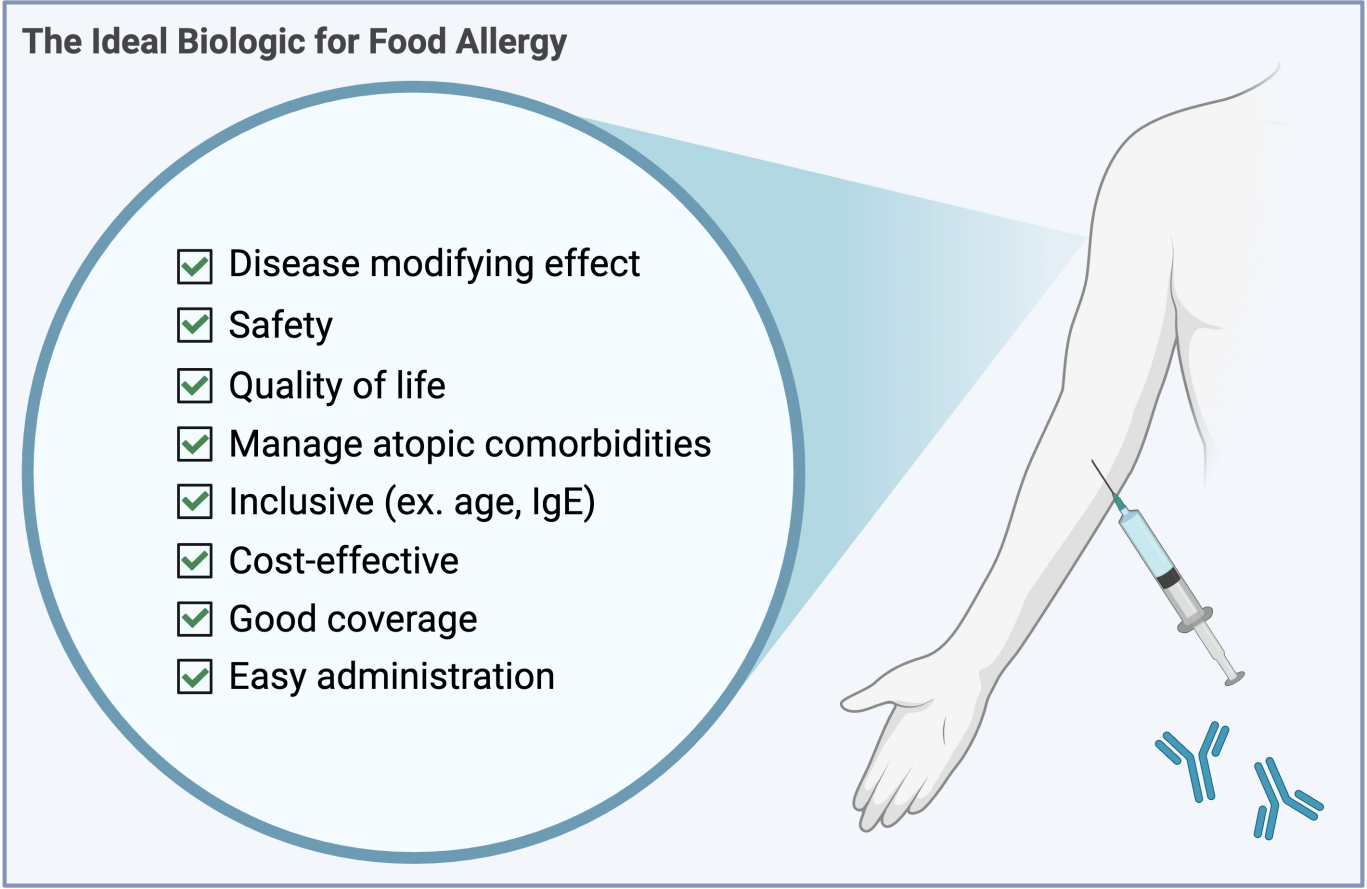
A checklist for the ideal front-line biologic to treat food allergy.

Due to the immune modulating properties of these drugs on type 2 pathways, there is potentially an increased risk of parasite susceptibility and hypersensitivity. Given these limitations, the use of therapeutics such as omalizumab must be strongly weighed against alternatives such as AIT for first-line treatment and consider patient factors including age, dosing, duration, comorbidities, cost, and availability (**Figure 1**). A monoclonal such as dupilumab that has the potential to be disease modifying and attenuate the atopic march would change the risk-benefit considerations, with meta-analyses highlighting the importance of further investigations (51).

## 5 Future directions and research gaps

Determining the appropriate use cases of biologics for food allergy requires further research as there are poor predictors of persistence of allergy and of severe reactions to elucidate who might benefit most from this approach. Head-to-head studies are required to stratify an approach where monoclonal antibodies might be best utilized in conjunction with AIT vs AIT alone, or as a monotherapy, especially given a wide variety in protocols between studies. There is an immense need for real-world data on dose optimization which currently models other atopic conditions, long-term outcomes (sustained unresponsiveness, cost-utility), and duration of treatment. These studies will aid in designing considerations for optimizing biologics for food allergy, especially in the setting of alternative treatment strategies where omalizumab may not receive approval in other countries as a first-line modality. Overall, the exploration of new effective biologic targets beyond omalizumab may also provide insight into disease modifying therapies in the context of other atopic diseases (e.g. dupilumab) and adapting treatment approaches to food allergy.

## 6 Conclusions

Longterm goals for food allergy management should include disease-modifying therapies, given the immense burden to patients. The future of monoclonal antibody use will require dose optimization and stratified approaches, with an emphasis on judicious, individualized use, potentially in those who have failed disease modifying therapies and/or have high comorbidities. Given that low risk and effective therapies such as SLIT, EPIT, and lower doses for OIT are being increasingly validated, monoclonal antibodies and other very expensive management strategies should be strategically used and adapted to forthcoming clinical trials and have their first-line treatment with broad eligibility criteria reassessed with new therapeutic developments. For those with a food allergy, active and ideally early treatment are key to decreasing the overall food allergy burden. As disease modifying treatments evolve, some biologics may be best considered adjunctive or second-line and remain an exciting part of the allergist’s toolkit in this new era of the treatment of food allergy.

## Data Availability

The original contributions presented in the study are included in the article/supplementary material. Further inquiries can be directed to the corresponding author.
